# Association Aamong Ppolymorphisms in the Aapoptosis‐Rrelated NKX3‐1, Caspase‐3, Caspase‐9, and BCL‐2 Genes and Prostate Cancer Susceptibility From 9706 Cases and 12,567 Controls

**DOI:** 10.1002/cnr2.70206

**Published:** 2025-05-08

**Authors:** Yanyan Feng, Zhenting Feng, Dan Li, Jiandong Gui, Zhihong Song, Xiaohua Xie, Lijie Zhu, Yuanyuan Mi

**Affiliations:** ^1^ Department of Oncology Affiliated Hospital of Jiangnan University Wuxi China; ^2^ Wuxi Medical College, Jiangnan University Wuxi China; ^3^ Department of Nursing The First Affiliated Hospital of Shenzhen University/Shenzhen Second People's Hospital Shenzhen China; ^4^ Department of Urology Affiliated Hospital of Jiangnan University Wuxi China

**Keywords:** apoptosis‐related genes, BCL‐2, caspase‐3, caspase‐9, meta‐analysis, NKX3‐1, polymorphisms, prostate cancer

## Abstract

**Background:**

While there is a growing volume of evidence suggesting that relatively prevalent functional polymorphisms present within apoptosis‐related genes may influence human prostate cancer (PCa) susceptibility, the clinical relevance of these findings remains inconclusive.

**Aims:**

This meta‐analysis was thus developed with the goal of generating more precise estimates of the relationships between polymorphisms in four apoptosis‐associated genes (NKX3‐1, caspase‐3, caspase‐9, and BCL‐2) and the risk of PCa.

**Methods and Results:**

The PubMed, Web of Science, Google Scholar, Embase, Cochrane Library, and SinoMed (CNKI and Wanfang) databases were searched for relevant studies published through December 20, 2023, using the following keywords: “polymorphism” or “variant” and “carcinoma” or “cancer” or “tumor” and “NKX3‐1,” “CASP3” or “Caspase‐3,” “CASP9” or “Caspase‐9,” “BCL‐2” or “B‐cell lymphoma” and “prostate cancer” or “PCa” or “prostate adenocarcinoma.” This approach led to the identification of 22 case–control studies related to the association between apoptosis‐related gene polymorphisms and PCa susceptibility enrolling 9706 cases and 12 567 controls. Subsequent analyses revealed that the *NKX3‐1* rs2228013, *CASP9* rs1052571, and *CASP9* rs4645982 polymorphisms were associated with greater PCa risk, whereas the *CASP3* rs4647603 polymorphism was associated with a risk reduction.

**Conclusion:**

These findings provide strong evidence for the potential contributions of polymorphisms in the apoptosis‐related caspase‐3, caspase‐9, and NKX3‐1 genes in the onset and progression of PCa.

## 1 Introduction

Prostate cancer (PCa) is a leading cancer type among men, with millions of new diagnoses throughout the world each year [1]. In 2023, for example, the International Agency for Research on Cancer forecasts approximately 288,300 new PCa diagnoses and 34,700 related deaths at the global level [2]. PCa incidence rates rise with age such that this cancer type is most common among men over the age of 65 [3]. PCa incidence also varies as a function of ethnicity and geographical location, with Caribbean and African American males facing particularly high PCa rates, whereas this malignancy is less common among Asian men [4]. While Japan exhibits the lowest PCa incidence among Asian nations, rates of PCa diagnoses in China continue to rise annually [5, 6]. Positive family history has been increasingly established as an important factor associated with the risk of PCa [7, 8]. Genetic association studies seek to elucidate the genetic risk factors associated with particular cancers or other diseases of interest. Despite the high rates of PCa diagnoses in African American populations, however, members of this community remain underrepresented in genetic association studies focused on this form of cancer [9].

PCa develops through a multistage process that is influenced by a diverse array of factors, with both genetic and epigenetic changes playing essential roles in facilitating oncogenesis [10]. Research focused on the genetic epidemiology of PCa risk has revealed PCa to be the cancer type with the fourth highest risk of presenting in multiple members of a given family after lip melanoma, skin melanoma, and ovarian cancer [11]. A family history positive for PCa is thus regarded as a major risk factor for developing this form of malignancy [12], such that genetic factors are thought to serve as particularly important regulators of PCa incidence more so than in other human cancer types [13, 14]. Consistently, the variations in PCa diagnosis rates among regions and ethnic groups are thought to be partially attributable to differences in the prevalence of particular single nucleotide polymorphisms (SNPs) associated with PCa risk [15]. In particular, SNPs present within apoptosis‐associated genes have been shown to be closely tied to the odds of developing PCa [16, 17].

Apoptosis is an essential mechanism of programmed cell death that shapes both pathological and physiological processes [18, 19]. When apoptotic activity is dysregulated in eukaryotic cells, this can result in abnormal survival outcomes [20]. Accordingly, many types of malignant cells have developed mechanisms that enable them to evade apoptotic death through the modulation of pro‐ and anti‐apoptotic factors, thereby altering signaling pathways in the intracellular and extracellular environment to alter the expression of apoptosis‐associated genes [19]. The external and internal apoptotic pathways can become dysregulated in PCa, contributing to the ability of tumor cells to avoid undergoing apoptosis such that these malignant cells can instead proliferate and disseminate [18, 21].

The antiapoptotic protein B‐cell lymphoma‐2 (BCL‐2) has been a focus of intensive research efforts [22], as changes in BCL‐2 expression levels can contribute to improperly regulated apoptotic activity and aberrant cell proliferation conducive to oncogenesis and tumor progression [23]. The BCL‐2 oncogene is located on the mitochondrial membrane and has been clearly shown to be a key factor in carcinogenesis and resistance to drug‐induced apoptosis in different types of adenocarcinoma [24]. The prospective PROCAGENE study, which enrolled 702 patients with PCa, found that overall survival (OS) in patients with PCa was associated with the homozygous *BCL2*‐938 CC genotype [25]. In line with this evidence regarding the importance of *BCL2* SNPs in PCa, Hirata et al. also emphasized the association between apoptosis‐related gene polymorphisms and oncogenic risk [26], while Souza et al. [27] found that the *BCL2*‐938 C > A polymorphism was related to an elevated risk of biochemical recurrence following radical prostatectomy, in addition to serving as an independent predictor of both OS and recurrence‐free survival (RFS).

High levels of NKX3‐1 protein expression are detectable within prostate epithelial cells, wherein it functions to preserve prostate specification and to support prostate ductal stem cell maintenance while inhibiting inflammation, DNA damage, and PCa incidence through its ability to shield the mitochondria from oxidative injury [28]. Given its ability to tightly regulate prostate epithelial cell proliferation and differentiation [29], NKX3‐1 is regarded as the most important tumor suppressor protein related to PCa incidence [30, 31]. Chen et al. [32] demonstrated that a particular SNP (rs21687) within the site where NKX3‐1 binds to the promoter of the gene encoding L‐plastin may contribute to a lower risk of PCa incidence. Martinez et al. [33] further demonstrated that polymorphic NKX3‐1 alleles can encode an abnormal version of this protein with altered DNA binding activity that can impact the risk of PCa incidence.

Caspase‐3 and caspase‐9 are firmly established as important enzymatic mediators of apoptotic cell death [34]. Caspase‐3 functions as the final executioner caspase within the apoptotic pathway following its activation in response to pro‐apoptotic signals such that it can cleave substrate proteins to ensure the progression of the apoptotic cascade [35]. Caspase‐9, in contrast, serves as an upstream initiator of apoptotic death through its interactions with cytochrome c and apoptosis protease‐activating factor 1, activating the apoptotic cascade [36]. By catalyzing this signaling and the downstream activation of caspase‐3 and other enzymes, caspase‐9 can thus promote the clearance of abnormal cells to help maintain systemic homeostasis [37]. Mittal et al. [38] reported that the *CASP3* rs4647603 CT genotype and T allele were related to an elevated risk of PCa in individuals who are obese, with a positive correlation between caspase‐3 and such cancer risk. Souza et al. [16] further identified an association between *CASP3, NKX3‐1*, and *BCL2* gene polymorphisms and PCa risk. These results emphasize the close association between these particular SNPs and PCa, suggesting that they may offer value as molecular biomarkers that can guide the prognostic evaluation of patients with this form of cancer.

Prior studies have explored the utility of particular SNPs as molecular markers associated with PCA patient outcomes [39, 40]. The combined analysis of SNPs in the *NKX3‐1, CASP3, CASP9*, and *BCL2* genes has been tied to adverse PCa patient outcomes [16, 25], suggesting the utility of these SNPs as prognostic biomarkers in this context. However, additional studies will be essential to validate the relevance of these findings and to assess the clinical relevance of these biomarkers. The present meta‐analysis was thus conducted with the goal of comprehensively screening published studies in order to facilitate pooled analyses aimed at objectively clarifying the link between apoptosis‐related gene polymorphisms and PCa risk. The results of this study will provide an evidence‐based foundation for early screening and clinical management strategies for PCa patients.

## 2 Materials and Methods

### 2.1 Study Search Process

The PubMed, Web of Science, Google Scholar, Embase, Cochrane Library, and SinoMed (CNKI and Wanfang) databases were searched for relevant studies published through October 20, 2023, using the following keywords: “polymorphism” or “variant” and “carcinoma” or “cancer” or “tumor” and “NKX3‐1,” “CASP3” or “Caspase‐3,” “CASP9” or “Caspase‐9,” “BCL‐2” or “B‐cell lymphoma” and “prostate cancer” or “PCa” or “prostate adenocarcinoma.” There were no limitations placed on language or year of publication.

### 2.2 Study Selection

Studies eligible for inclusion in the present meta‐analysis were those that: (i) focused on the association between specific apoptosis‐associated gene polymorphisms and PCa risk, (ii) were case–control studies, and (iii) provided sufficient genotype numbers in the case and control groups. Studies were excluded if they (i) did not include a control population, (ii) did not provide complete information on genotype frequencies, (iii) were duplicates, (iv) were meta‐analyses, (v) were clinical trials, (vi) focused on other polymorphisms, or (vii) were systematic reviews.

### 2.3 Data Extraction

Two investigators (YF and ZF) independently selected relevant studies and used a standardized approach to extract the following information from each study: first author, publication year, country, study population ethnicity, control population source, total case and control numbers, apoptosis‐related genes of interest, polymorphisms that were analyzed, numbers of genotypes and alleles, Hardy–Weinberg equilibrium (HWE) results for control subjects, and the genotyping methods employed.

### 2.4 Statistical Analyses

Initially, data related to SNPs in apoptosis‐related genes including NKX3‐1 (rs2228013, rs2228013, rs11781886, and rs1512268), caspase‐3 (rs4647603), BCL‐2 (rs2279115), and caspase‐9 (rs1052571, rs4645978, rs4645982) were extracted, with data for each SNP being extracted from two or more studies. The ethnicity of participants in these studies was classified using two different classification patterns (Asian, Caucasian, African, and South American) based on source analyses of control subgroups, including hospital‐based (HB) and population‐based (PB) classifications. Genotypic distributions in cases and controls were used to compute odds ratio (OR) values with 95% confidence intervals (CIs) as a means of clarifying the association between these apoptosis‐related gene polymorphisms and PCa incidence. These ORs were analyzed with Z‐tests, and heterogeneity was evaluated with the Q‐test, with *p* < 0.05 as the threshold used to define significant heterogeneity. Fixed‐effects models were used unless significant heterogeneity was observed, in which case a random‐effects model was instead employed. Allelic contrast (M‐allele vs. W‐allele), heterozygous (MW vs. WW), homozygous (MM vs. WW), dominant (MM + MW vs. WW), and recessive (MM vs. MW + WW) genetic models were then employed to examine the associations between particular SNPs and susceptibility to PCa. Pearson's chi‐square test was used to assess the HWE status of control subjects, with *p* < 0.05 as the threshold used to define significance. Publication bias was assessed based on Egger's regression test and Begg's funnel plots. Stata 11.0 (StataCorp LP, TX, USA) was used to perform all statistical analyses.

### 2.5 Bioinformatics Analyses

GEPIA (http://gepia.cancer‐pku.cn/) was used to evaluate CASP3 and NKX3‐1 expression in PCa tumors and adjacent tissues and to evaluate patient disease‐free survival (DFS) with NKX3‐1. Similarly, the Cancer Genome Atlas (TCGA) database (https://www.cancer.gov/ccg/research/genome‐sequencing/tcga) was used to compare BCL‐2 expression levels in tumors and normal tissues. The STRING database (http://string‐db.org/) was also used to construct gene–gene interaction networks for each of these four apoptosis‐associated genes in order to better understand how they may contribute to PCa risk.

## 3 Results

### 3.1 Study Characteristics

**FIGURE 1:**
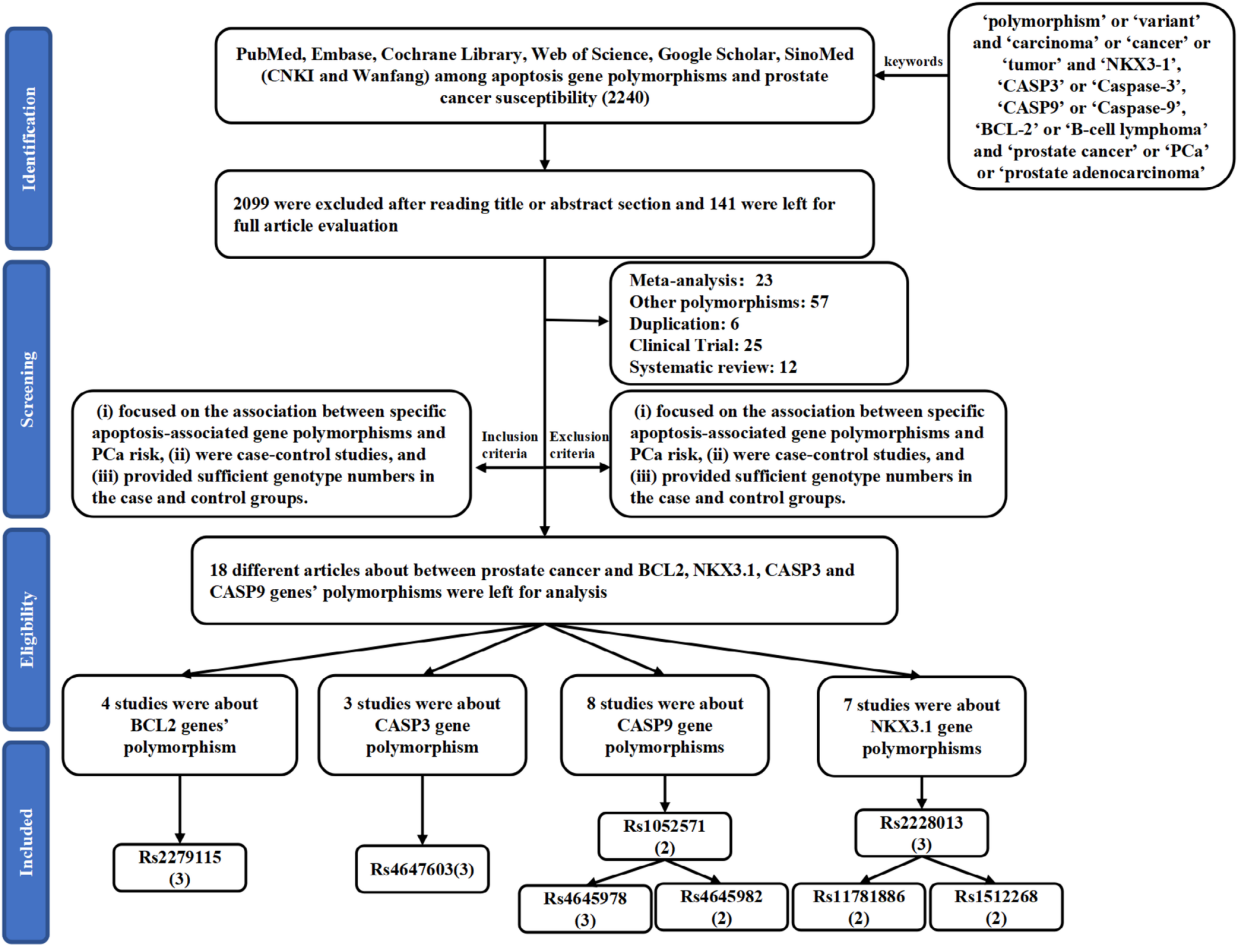
The flowchart illustrating the search strategy used effectively to identify association between apoptosis‐related genes and prostate cancer risk.

**FIGURE 2:**
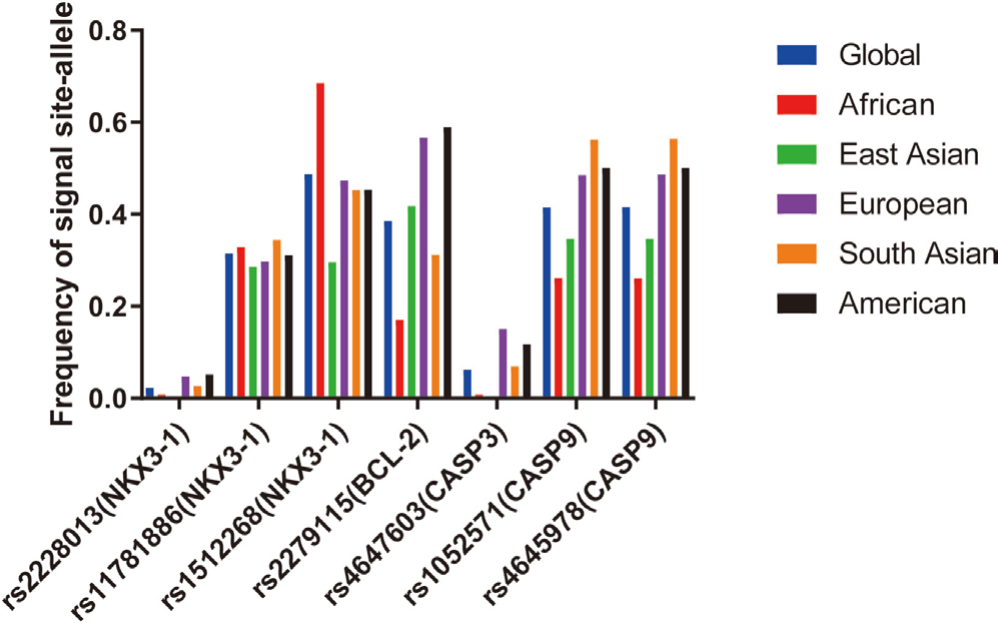
1000 Genomes database results corresponding to the frequencies of signal site alleles in the *NKX3‐1* (rs2228013, rs11781886, and rs1512268), *CASP3* (rs4647603), *BCL2* (rs2279115), and *CASP9* (rs1052571 and rs4645978) genes.

**FIGURE 3:**
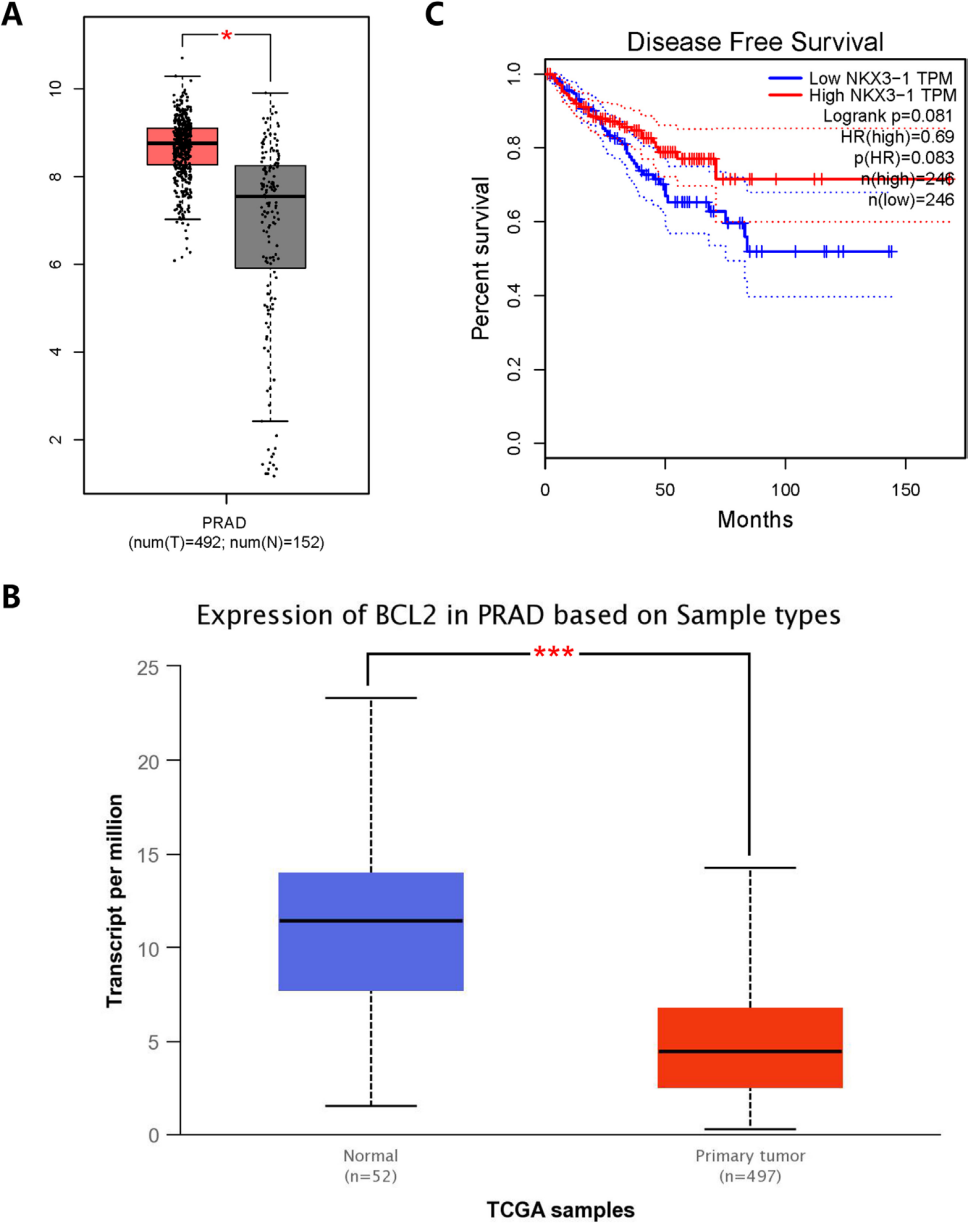
(A) The expression of the *CASP3* gene in PCa. (B) Analyses of disease‐free survival (DFS) for patients with PCa, revealing a positive correlation between *NKX3‐1* expression and patient DFS. (C) *BCL2* expression in PCa cases in the TCGA database with different sample types. PCa: Prostate adenocarcinoma. TCGA: The Cancer Genome Atlas.

An initial literature search revealed 2240 potentially relevant articles, of which 2099 were excluded following title or abstract review. Of the remaining articles, 141 were excluded because they were duplicates (*n* = 6), meta‐analyses (*n* = 23), clinical trials (*n* = 25), systematic reviews (*n* = 12), or focused on other polymorphisms (*n* = 57). The remaining articles included 20 reports focused on associations between SNPs in these four apoptosis‐related genes of interest (*NKX3‐1, CASP3, CASP9*, and *BCL2*) and PCa. These included three articles related to *BCL2* polymorphisms focused on rs2279115 were analyzed, as well as three focused on the *CASP3* rs4647603 SNP, seven focused on *CASP9* SNPs (including two focused on rs1052571, three focused on rs4645978, and two focused on rs4645982 that were retained for analysis), and seven focused on *NKX3‐1* SNPs (including three focused on rs2228013, two focused on rs1178188, and two focused on rs1512268). The basic characteristics of these studies are summarized in Table 1, including the first author, publication year, country, ethnicity, control population source, numbers of cases and controls, apoptosis‐related genes of interest, polymorphisms of interest, numbers of genotypes and alleles, HWE results, and genotyping methodology. In total, these case–control studies included 9706 cases and 12,567 controls (Figure 1), with control subjects primarily being derived from healthy populations. Overall, these analyses included 3 Caucasian, 9 Asian, 4 South American, and 4 American case–control studies, of which 11 and 9 were respectively based on HB and PB populations. Next, the 1000 Genomes Browser was used to assess the minor allele frequency (MAF) values for rs2228013 (*NKX3‐1*), rs11781886 (*NKX3‐1*), rs1512268 (*NKX3‐1*), rs4647603 (*CASP3*), rs2279115 (*BCL2*), rs1052571 (*CASP9*), and rs4645978 (*CASP9*) in six major global populations (https://www. ncbi.nlm.nih.gov/snp/rs2228013\rs11781886\rs1512268\rs4647603\rs2279115\rs1052571\rs4645978) (Figure 2). Based on HWE values, 18 articles were eligible for inclusion in pooled analyses. The TCGA database revealed that *CASP3* expression in PCa tumor samples was elevated relative to normal tissues (*p* < 0.05) (Figure 3A), whereas *BCL2* was downregulated in PCa tumors (*p* < 0.05) (Figure 3B). PCa patients expressing higher *NKX3‐1* levels also trended toward exhibiting better DFS outcomes relative to patients expressing lower levels of this tumor suppressor gene (Figures 3C).

**TABLE 1:** Characteristics of studies of polymorphisms in apoptosis‐related genes and PCa risk included in our meta‐analysis.

First author	Year	Origin	Ethnicity	SOC	Case	Control	Gene	SNP	Case			Control			HWE	Method
									MM	MW	WW	MM	MW	WW		
Muhlbradt	2013	USA	America	PB	937	1086	NKX3‐1	rs2228013	2	91	844	4	84	998	0.125	RT‐PCR
Martinez	2014	USA	America	PB	1845	3102	NKX3‐1	rs2228013	2	150	1693	5	233	2864	0.981	TaqMan
Gelmann	2002	USA	America	PB	558	695	NKX3‐1	rs2228013	2	57	499	3	55	637	0.133	TaqMan
Souza	2022	Brazil	South America	HB	283	283	NKX3‐1	rs11781886	31	117	135	26	104	153	0.18	TaqMan
Martinez	2014	USA	America	PB	1857	3112	NKX3‐1	rs11781886	136	737	984	211	1184	1717	0.722	TaqMan
Hui	2014	China	Asian	HB	285	287	NKX3‐1	rs1512268	118	139	28	131	133	23	0.176	PCR‐HRM
Hui	2012	China	Asian	HB	124	137	NKX3‐1	rs1512268	14	67	43	11	65	61	0.267	PCR‐HRM
López‐Trigo	2018	Spain	Caucasian	PB	242	191	Caspase‐3	rs4647603	4	77	161	4	57	130	0.433	MALDI‐TOF
Mittal	2012	India	Asian	HB	192	225	Caspase‐3	rs4647603	89	76	27	129	80	16	0.462	PCR‐RFLP
Souza	2022	Brazil	South America	HB	283	283	Caspase‐3	rs4647603	4	73	206	11	67	205	0.073	TaqMan
Hirata	2009	Japan	Asian	HB	140	167	BCL‐2	rs2279115	31	58	51	30	90	47	0.249	PCR‐RFLP
Souza	2022	Brazil	South America	HB	283	283	BCL‐2	rs2279115	79	138	66	75	130	78	0.172	TaqMan
Meyer	2012	USA	Caucasian	PB	509	466	BCL‐2	rs2279115	104	259	146	76	238	152	0.286	TaqMan
Lavender	2012	USA	Caucasian	PB	1162	1111	Caspase‐9	rs1052571	253	589	320	210	553	348	0.711	PCR‐MDR
Souza	2022	Brazil	South America	HB	283	283	Caspase‐9	rs1052571	71	151	61	83	132	68	0.277	PCR‐MDR
Altamemi	2020	IRAQ	Asian	HB	50	50	Caspase‐9	rs4645978	12	22	16	5	12	33	0.033	ARMS‐PCR
Kesarwani	2010	India	Asian	HB	173	198	Caspase‐9	rs4645978	49	42	82	47	81	70	0.016	PCR‐RFLP
George	2012	India	Asian	PB	165	205	Caspase‐9	rs4645978	48	40	77	47	83	75	0.012	PCR‐RFLP
George	2012	India	Asian	PB	165	205	Caspase‐9	rs4645982	27	95	43	40	121	44	0.009	PCR‐RFLP
Kesarwani	2010	India	Asian	HB	170	198	Caspase‐9	rs4645982	29	101	40	40	121	37	0.001	PCR‐RFLP

Abbreviations: ARMS‐PCR, amplification refractory mutation system‐polymerase chain reaction; HB, hospital based; HWE, Hardy–Weinberg equilibrium of control group; MALDI‐TOF, matrix‐assisted laser desorption ionization time‐of‐flight; NA, not available; PB, population based; PCR‐CTPP, PCR‐confronting two‐pair primers; PCR‐HRM, polymerase chain reaction by high‐resolution melting curves; PCR‐MDR, polymerase chain reaction by multifactor dimensionality reduction; RT‐PCR, real‐time PCR; SOC; source of control; PCR‐RFLP, polymerase chain reaction followed by restriction fragment length polymorphism.

### 3.2 Meta‐Analysis

**FIGURE 4:**
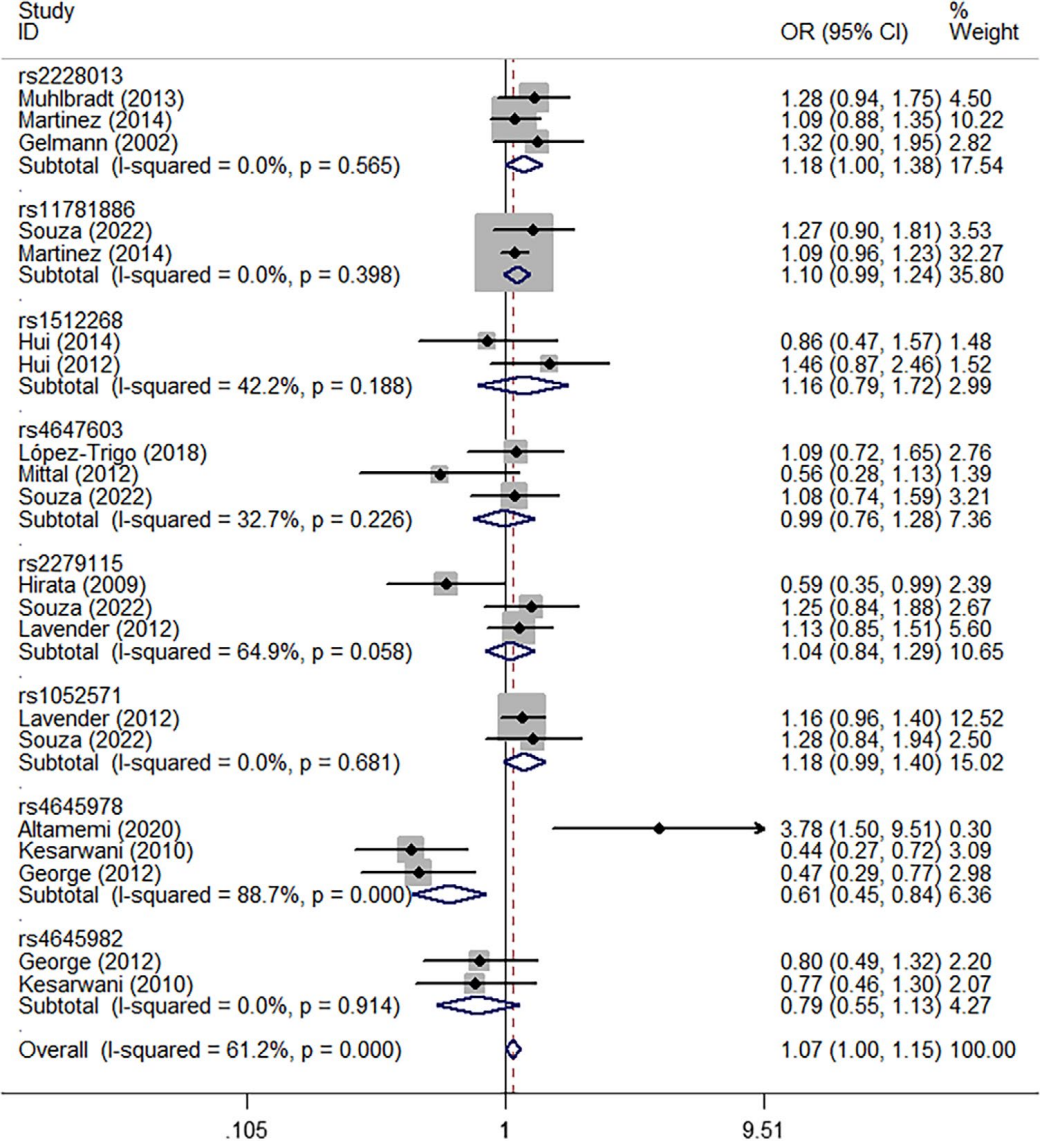
A forest plot representing the relationship between the NKX3‐1 rs2228013 polymorphism and PCa risk (GA vs. AA model).

**FIGURE 5:**
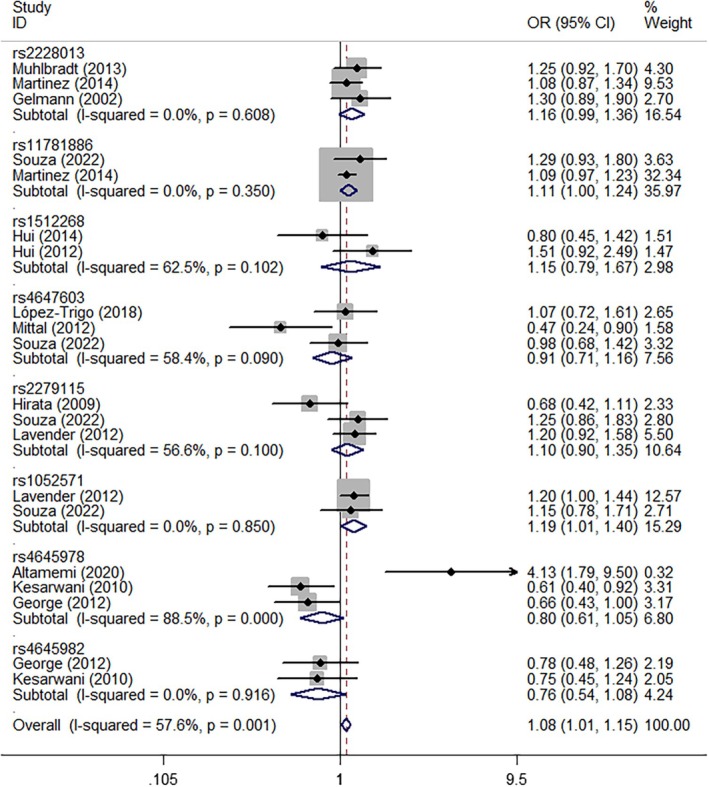
A forest plot representing the relationship between the CASP9 rs1052571 polymorphism in PCa (GG+GA vs. AA model).

**FIGURE 6:**
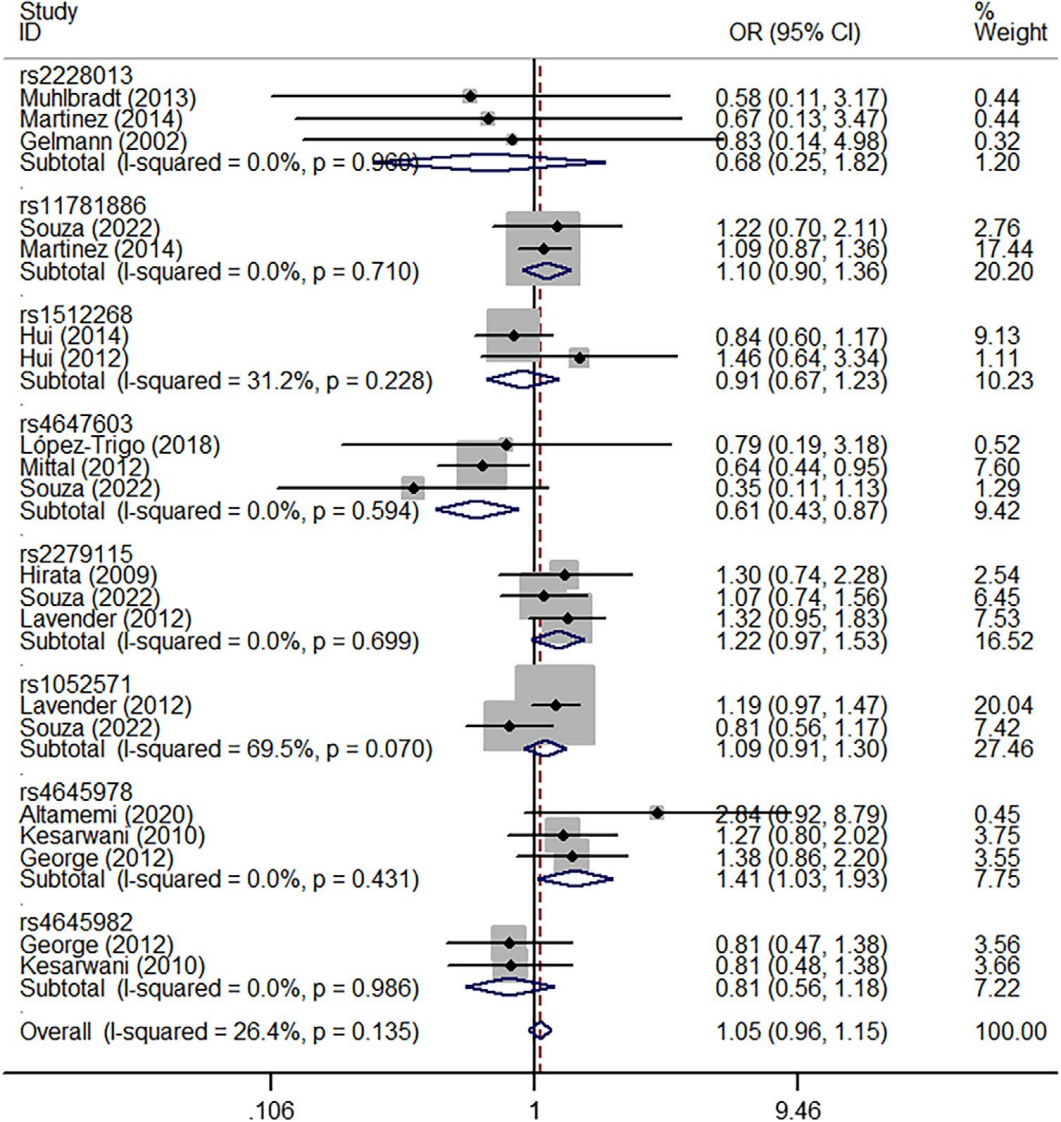
A forest plot representing the relationship between the CASP9 rs4645978 polymorphism in PCa (GG vs. GA+AA model).

**FIGURE 7:**
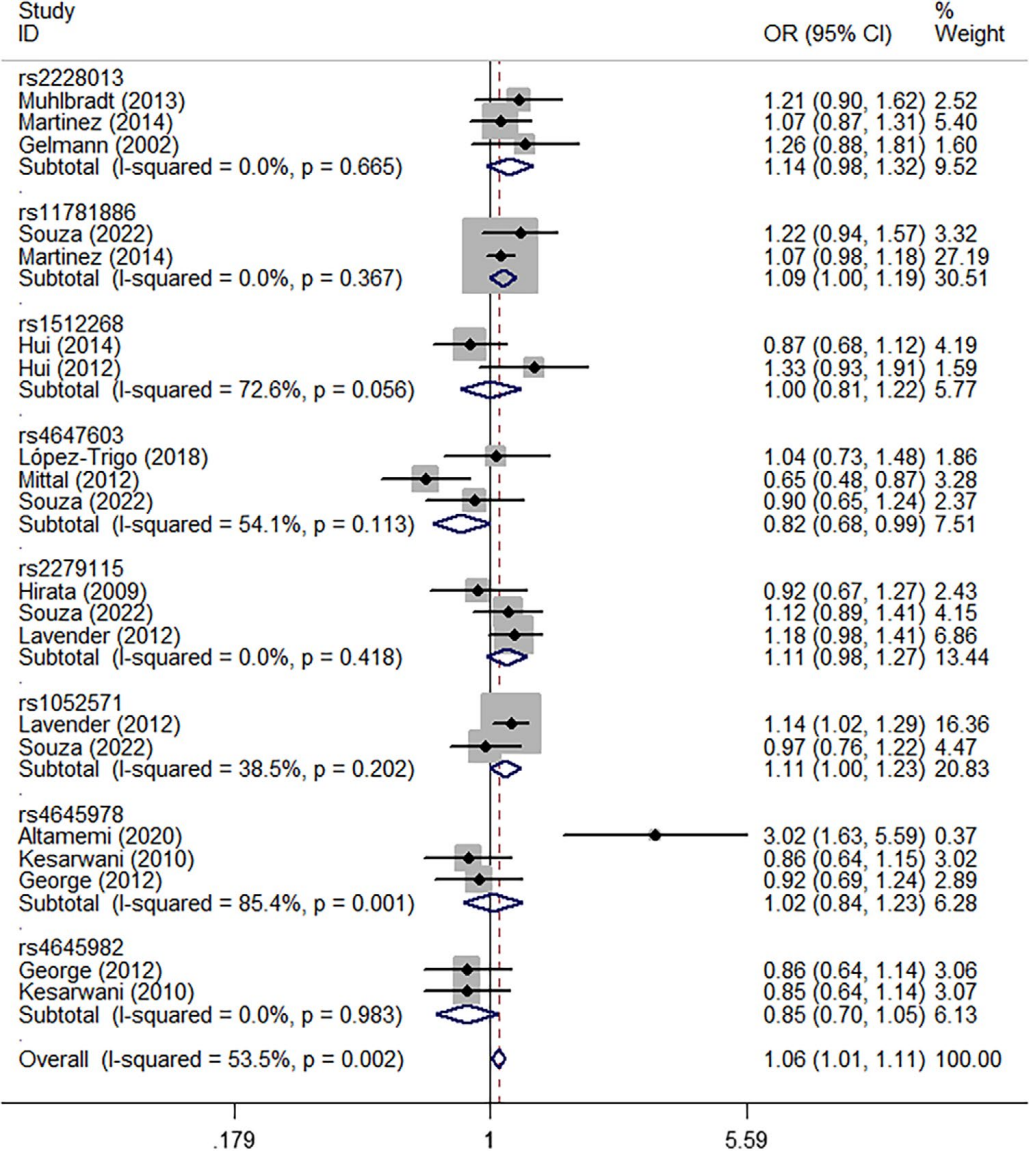
A forest plot representing the relationship between the CASP3 rs4647603 polymorphism in PCa (G‐allele vs. A‐allele model).

In pooled analyses, *NKX3‐1* rs2228013 (GA vs. AA, OR = 1.18, 95% CI = 1.00–1.38, *P*_heterogeneity_ = 0.565, *p* = 0.047, Figure 4), *CASP9* rs1052571 (GG + GA vs. AA, OR = 1.19, 95% CI = 1.01–1.40, *P*_heterogeneity_ = 0.850, *p* = 0.037, Figure 5), and *CASP9* rs4645982 (GG vs. GA + AA, OR = 1.41, 95% CI = 1.03–1.93, *P*_heterogeneity_ = 0.431, *p* = 0.032, Figure 6) were associated with an elevated risk of PCa when evaluated using different genetic models. Conversely, *CASP3* rs4647603 was associated with a significant reduction in PCa risk (GG vs. AA, OR = 0.44, 95% CI = 0.26–0.75, *P*_heterogeneity_ = 0.647, *p* = 0.002; GG vs. GA + AA, OR = 0.61, 95% CI = 0.43–0.87, *P*_heterogeneity_ = 0.594, *p* = 0.006; G‐allele vs. A‐allele, OR = 0.82, 95% CI = 0.68–0.99, *P*_heterogeneity_ = 0.113, *p* = 0.041, Figure 7) (Table 2).

**TABLE 2:** Stratified analysis of polymorphisms in apoptosis genes and PCa susceptibility.

Variables	*N*	Case/control	M‐allele vs. W‐allele	MW vs. WW	MM vs. WW	MM + MW vs. WW	MM vs. MW + WW
			OR(95% CI) *p*_h_ *p*	OR (95% CI) *p*_h_ *p*	OR (95% CI) *p*_h_ *p*	OR (95% CI) *p*_h_ *p*	OR (95% CI) *p*_h_ *p*
rs2228013 (NKX3‐1)	3	3340/4883	1.14 (0.98–1.32) 0.665 0.099	1.18 (1.00–1.38) 0.565 0.047	0.69 (0.26–1.85) 0.958 0.462	1.16 (0.99–1.36) 0.068 0.066	0.68 (0.25–1.82) 0.960 0.441
rs11781886 (NKX3‐1)	2	2140/978	1.09 (1.00–1.19) 0.367 0.056	1.10 (0.99–1.24) 0.398 0.087	1.15 (0.93–1.43) 0.558 0.186	1.11 (1.00–1.24) 0.350 0.056	1.10 (0.90–1.36) 0.710 0.348
rs1512268 (NKX3‐1)	2	409/424	1.06 (0.70–1.60) 0.056 0.797	1.16 (0.79–1.72) 0.188 0.444	0.99 (0.60–1.61) 0.102 0.955	1.15 (0.79–1.67) 0.102 0.465	0.91 (0.67–1.23) 0.228 0.538
rs4647603 (CASP3)	3	717/699	0.82 (0.68–0.99) 0.113 0.041	0.99 (0.76–1.28) 0.226 0.929	0.44 (0.26–0.75) 0.647 0.002	0.85 (0.57–1.28) 0.090 0.444	0.61 (0.43–0.87) 0.594 0.006
rs2279115 (BCL‐2)	3	932/916	1.11 (0.96–1.27) 0.418 0.105	0.98 (0.67–1.45) 0.058 0.939	1.27 (0.98–1.65) 0.563 0.072	1.10 (0.90–1.35) 0.100 0.343	1.22 (0.97–1.53) 0.699 0.083
rs1052571 (CASP9)	2	1445/1394	1.11 (1.00–1.23) 0.202 0.060	1.18 (0.99–1.40) 0.681 0.065	1.23 (0.99–1.52) 0.236 0.058	1.19 (1.01–1.40) 0.850 0.037	1.01 (0.69–1.46) 0.070 0.945
rs4645978 (CASP9)	3	388/453	1.24 (0.71–2.17) 0.001 0.450	0.85 (0.30–2.36) 0.000 0.755	1.32 (0.65–2.70) 0.033 0.439	1.08 (0.45–2.62) 0.000 0.857	1.41 (1.03–1.93) 0.431 0.032
rs4645982 (CASP9)	2	335/403	0.85 (0.70–1.05) 0.983 0.132	0.79 (0.55–1.13) 0.914 0.195	0.68 (0.43–1.08) 0.950 0.101	0.76 (0.54–1.08) 0.916 0.125	0.81 (0.56–1.18) 0.986 0.273

*Note:* *p*_h_: value of *Q*‐test for heterogeneity test; *p*: *Z*‐test for the statistical significance of the OR.

### 3.3 Gene–Gene Interaction Network Analyses

**FIGURE 8:**
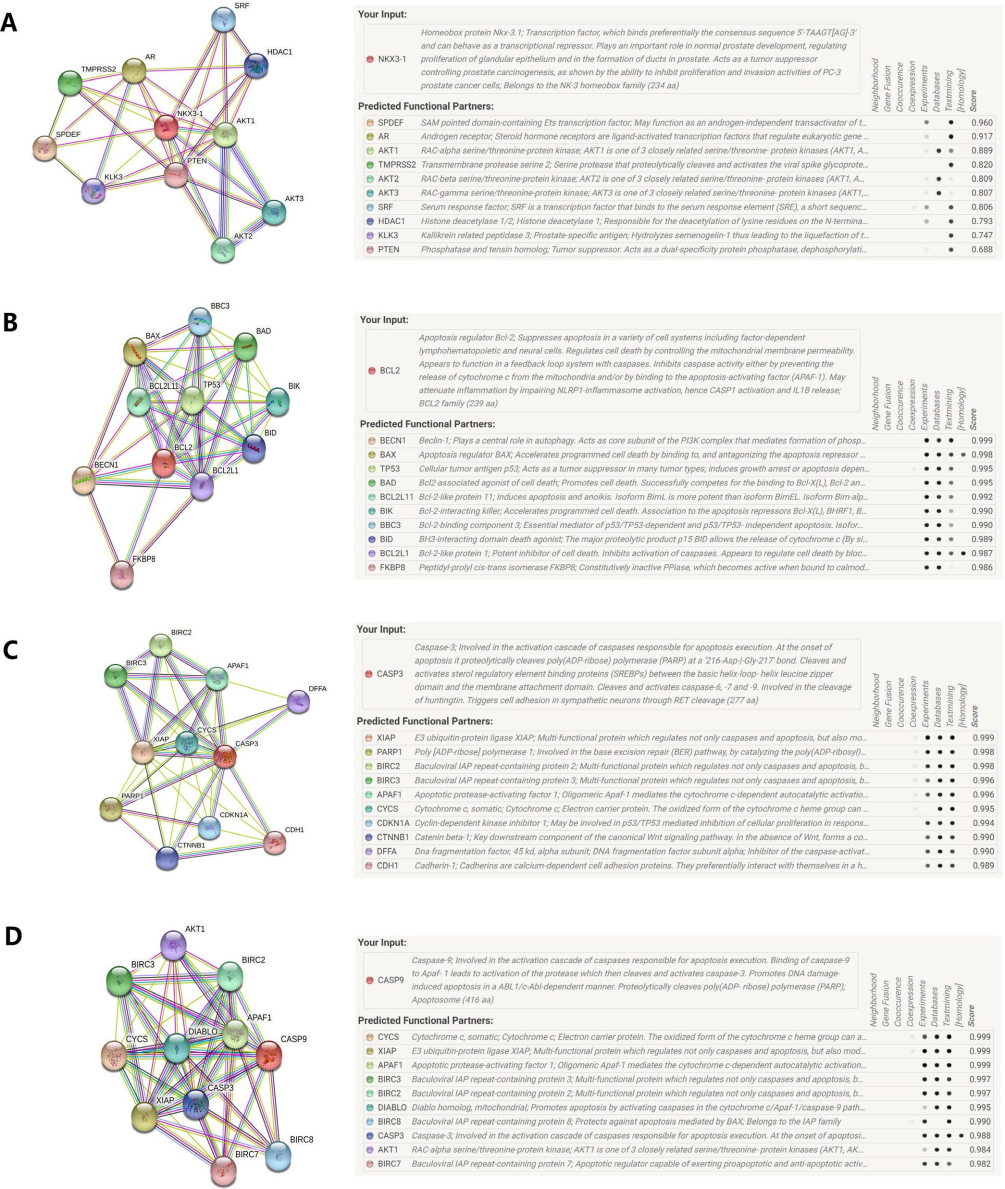
(A–D) Networks documenting interactions between NKX3‐1 (A), caspase‐3 (B), BCL‐2 (C), and caspase‐9 (D) and other genes as retrieved using the STRING database, with details provided for 10 core genes in each case.

In order to better understand the role of apoptotic genes in prostate cancer, the STRING database was next used to characterize the interactions among NKX3‐1, caspase‐3, BCL‐2, caspase‐9, and a variety of other genes (Figure 8). The genes that were most closely associated with NKX3‐1 included SAM‐pointed domain‐containing Ets transcription factor (*SPDEF*), androgen receptor (*AR*), RAC‐alpha serine/threonine protein kinase (*AKT1*), transmembrane protease serine 2 (*TMPRSS2*), and RAC‐beta serine/threonine protein kinase (*AKT2*). The genes most closely associated with caspase‐3 included the E3 ubiquitin ligase *XIAP*, poly [ADP‐ribose] polymerase 1 (*PARP1*), and baculoviral IAP repeat‐containing protein 2 (*BIRC2*). The genes most closely associated with BCL‐2 included beclin‐1, the apoptosis regulator BAX, and the antitumor protein p53 (*TP53*). The genes most closely associated with caspase‐9 included *CYS* (Cytochrome c, somatic\Cytochrome c\Electron carrier protein), *XIAP*, and apoptotic protease‐activating factor 1 (*APAF1*).

## 4 Discussion

PCa is among the most common cancers in the world, but many affected patients experience progressive disease and fail to attain persistent benefits from therapeutic interventions [41]. Apoptosis is an important target of cancer treatment efforts given the important role that this process plays in restraining the proliferation of malignant or injured cells [42]. Members of the BCL‐2 gene family function as key regulators of apoptotic activity with the potential to be leveraged to enable the more effective treatment of PCa and other forms of cancer [43]. Many prior reports have also documented key roles for caspase‐3, caspase‐9, and NKX3‐1 as regulators of apoptotic death, particularly in the context of treating PCa [44, 45].

Several prior reports have focused on polymorphisms in the genes encoding BCL‐2, caspase‐3, caspase‐9, and NKX3‐1, and their relevance to cancer treatment. For example, Javid et al. [46] pointed out that the BCL‐2 (*−938 CC*) genotype was an independent poor prognostic factor in patients with non–small cell lung cancer. Furthermore, in vitro studies of PCa cell lines (from androgen‐insensitive and androgen‐sensitive tumors) have shown that drug‐induced reductions in prostatic inflammation are associated with tumor apoptosis and death through specific mitochondrial pathways involving BCL‐2 [47]. The *CASP3* rs4647601 TT genotype has also been linked to head and neck squamous cell carcinoma risk [48], while *CASP9* rs4645981C is related to lung cancer incidence [49]. Gelmann et al. [42] highlighted the association between particular *NKX3‐1* allelic mutations and PCa risk. These data emphasize the relevance of apoptosis‐associated gene polymorphisms and cancer risk. Systematic studies focused on such polymorphisms in PCa and other cancer types, however, are lacking.

Through pooled analyses of prior case–control studies, the present data highlight the clinical relevance of specific SNPs in apoptosis‐related genes to PCa risk. Stratified analyses revealed that the *NKX3‐1* rs2228013, *CASP9* rs1052571, *CASP9* rs4645982, and *CASP3* rs4647603 SNPs were all closely tied to the risk of this cancer type. Specifically, the former three of these polymorphisms were identified as risk factors for PCa, whereas *CASP3* rs4647603 was a protective factor associated with a lower risk of disease.

Dysregulated apoptotic activity is a key factor that contributes to the inability of malignant cells to undergo appropriate programmed cell death in PCa tumors [50]. The present data highlight the close relationship between multiple apoptosis‐associated genes and cancer‐related outcomes, in line with the ability of SNPs in these genes to alter apoptotic signaling activity and thereby influence PCa incidence and progression. Members of the BCL‐2 gene family, for example, are well established as important regulators of apoptotic death [23], with BCL‐2 overexpression contributing to the development of this cancer type. Particular SNPs in these genes can contribute to altered signaling that enables transformed cells to more readily grow and metastasize. Efforts focused on studying SNPs in apoptosis‐associated genes thus have the potential to inform research focused on the mechanistic drivers of PCa incidence while also allowing for better predictions of disease susceptibility on an individualized basis and facilitating personalized treatment planning. Research analyzing SNP distributions in various populations can also help clarify the genetic heterogeneity of PCa risk, thereby uncovering new therapeutic targets and approaches to preventing or treating this devastating disease.

There are certain limitations to this meta‐analysis. First, two of the included studies failed to conform to the HWE. Gene–environment interactions and a range of other covariates have the potential to influence the findings of these pooled analyses, emphasizing a need for future comprehensive research efforts aimed at fully clarifying the association between particular genes and PCa‐related risk. There are also many apoptosis‐related genes that were not included in this meta‐analysis, such as the *BRAC* gene [51]. Continuing to pay attention to this aspect and making more perfect supplements is what our research group needs to improve. Even so, the present results offer greater statistical power than that afforded by any individual analysis, and the employed selection criteria were strict such that high‐quality case–control studies were used as a basis for these analyses, ensuring that the results are more robust and reliable.

## 5 Conclusion

In summary, the results of the present meta‐analysis suggest that certain SNPs in the genes encoding the apoptosis‐related proteins NKX3‐1, caspase‐3, caspase‐9, and BCL‐2 are associated with the risk of PCa. Future large‐scale research efforts, however, will be vital to fully confirm the role of these and other apoptosis‐related gene polymorphisms in shaping PCa onset and progression.

## Author Contributions

Y.F., Z.F., and D.L. were major contributors in writing the manuscript. Y.F. and Z.F. created all the figures. D.L. and J.G. performed the literature search. L.Z., X.X., and Y.M. made substantial contributions to the design of the manuscript and revised it critically for important intellectual content. All authors have read and approved the final version of this manuscript.

## Ethics Statement

The authors have nothing to report.

## Conflicts of Interest

The authors declare no conflicts of interest.

## Supporting information


**Table S1.** The patient's treatment methods, second tumors, comorbidity, histopathology, and PSA level were included.

## Data Availability

The data that support the findings of this study are available from the corresponding author upon reasonable request.
