# Supplementary material for: Association Aamong Ppolymorphisms in the Aapoptosis‐Rrelated NKX3‐1, Caspase‐3, Caspase‐9, and BCL‐2 Genes and Prostate Cancer Susceptibility From 9706 Cases and 12,567 Controls
Source: Cancer Rep (Hoboken). 2025 May 8;8(5):e70206. doi: 10.1002/cnr2.70206 (PMC12062516; doi:10.1002/cnr2.70206)
Supplement: Supplementary file 1 — Table S1. The patient's treatment methods, second tumors, comorbidity, histopathology, and PSA level were included. [file CNR2-8-e70206-s001.docx]

**Supplemental table 1** The patient's treatment methods, second tumors, comorbidity, histopathology, PSA level were included.

| **First author** | **Treatment methods** | **Second tumors** | **Comorbidity** | **Histopathology** | **PSA level** |
| --- | --- | --- | --- | --- | --- |
| Muhlbradt | Not mentioned | Not mentioned | Not mentioned | Yes | Not mentioned |
| Martinez | Not mentioned | Not mentioned | Not mentioned |  | Yes |
| Gelmann | Not mentioned | Not mentioned | Not mentioned | prostate cancer was diagnosed | |
| Souza | Not mentioned | Not mentioned | Not mentioned | Yes | Yes |
| Martinez | Not mentioned | Not mentioned | Not mentioned |  | Yes |
| Hui | Not mentioned | Not mentioned | Not mentioned | Yes | Yes |
| Hui | Not mentioned | Not mentioned | Not mentioned | Yes | Yes |
| López-Trigo | Not mentioned | Not mentioned | Not mentioned | prostate cancer was diagnosed | |
| Mittal | Not mentioned | Not mentioned | Not mentioned | Yes | Yes |
| Souza | Not mentioned | Not mentioned | Not mentioned | Yes | Yes |
| Hirata | Not mentioned | Not mentioned | Not mentioned | Yes | Yes |
| Souza | Not mentioned | Not mentioned | Not mentioned | Yes | Yes |
| Meyer | Not mentioned | Not mentioned | Not mentioned | Yes | Yes |
| Lavender | Not mentioned | Not mentioned | Not mentioned | Yes | Yes |
| Souza | Not mentioned | Not mentioned | Not mentioned | Yes | Yes |
| Altamemi | Not mentioned | Not mentioned | Not mentioned | prostate cancer was diagnosed | |
| Kesarwani | Not mentioned | Not mentioned | Not mentioned | Yes | Yes |
| George | Not mentioned | Not mentioned | Not mentioned | prostate cancer was diagnosed | Yes |
| George | Not mentioned | Not mentioned | Not mentioned | prostate cancer was diagnosed | Yes |
| Kesarwani | Not mentioned | Not mentioned | Not mentioned | Yes | Yes |
